# Summarization of Narrative Clinical Data of Inflammatory Bowel Disease With Foundational Large Language Models

**DOI:** 10.1016/j.gastha.2026.100967

**Published:** 2026-04-13

**Authors:** Soonwook Hong, Alexandra C. Greb, Prince Shah-Riar, Henry M. Zheng, Berkeley N. Limketkai

**Affiliations:** 1 Division of Gastroenterology, Icahn School of Medicine at Mount Sinai, New York, New York; 2 Division of Digestive Diseases, UCLA School of Medicine, Los Angeles, California; 3 Department of Internal Medicine, DHR Health, Edinburgh, Texas; 4 Department of Medical and Imaging Informatics, UCLA, Los Angeles, California

There is growing interest in applying large language models (LLMs) to summarize narrative clinical data. Recent studies have evaluated a wide range of inputs, from referral documents^1^ to inpatient progress notes.^2^ To date, few studies have evaluated LLMs in the specialty area of gastroenterology or subspecialty area of inflammatory bowel diseases (IBDs).

Evaluations of commercially available LLMs on clinical data have been limited by issues of data privacy. Open-source LLMs offer advantages here, with publicly available code and parameters, allowing for local implementations. The latest models have demonstrated performance comparable to privately owned LLMs such as OpenAI’s ChatGPT. Evaluating the performance of open-source models is critical for understanding future real-world applications.

In this study, we employed Meta’s open-source LLMs, Llama 2 and Llama 3, and OpenAI’s ChatGPT-4 and ChatGPT-4o to summarize clinical data from progress notes of simulated patients with IBD in the style of a standard “SOAP” (Subjective, Objective, Assessment, and Plan) note and evaluated the responses using a standardized Likert scale.

This study was conducted from August 16, 2023, to March 30, 2025. Analyses were performed with OpenAI’s ChatGPT-4 and ChatGPT-4o models and Meta’s Llama 2 and Llama 3.3 70 billion parameter models. Coauthors S.H. and P.S. drafted 11 “mock” progress notes containing common clinical scenarios encountered in the care of patients with IBD. Each progress note contained a “history of present illness” (HPI) section containing past medical history, relevant IBD history, information from previous endoscopic procedures, and current symptoms. HPIs were formatted in various ways, including using chronology, bulleted lists, and freeform paragraphs.

We utilized prompt chaining, a technique of breaking down a complex task into multiple, simpler tasks assigned to sequential prompts to achieve higher performance.^3^ The first prompt extracted clinically relevant data from the HPI of a single progress note. The output of the first prompt was incorporated into the second prompt, which instructed the LLM to generate a single-paragraph summary formatted in the style of a “SOAP” note assessment (Supplementary Figure 1). This was implemented with the Ollama application programming interface (v0.12.6).

Qualitative assessments of summarization are inherently difficult and depend on properties that are subjective and sometimes conflicting. We focused on separate evaluations of properties previously identified as being salient to a “good” summary.^4^ Assessments generated by the LLM and human authors were evaluated by 2 clinicians blinded to the identity of the “authors” for accuracy, thoroughness, relevancy, and fluency using a standardized rubric with a Likert scale ranging from 0 to 3 in each category (Supplementary Figure 1). The final score in each category was determined by averaging the 2 scores assigned by the physicians if they differed. Progress notes, summary outputs, and model scores are included as supplemental data. The final scores were compared across the different models with the Kruskal-Wallis test, and pairwise comparisons were performed using Dunn test. Statistical analyses were performed with R (The R Foundation, Vienna, Austria).

In our study, accuracy could vary significantly across models (H = 33.2; *P* < .01; Table 1), with summaries generated by the Llama 2-70B model being significantly less accurate than those written by human authors (2.0 [1.75] vs 3.0 [0]; *P* < .01). In contrast, summaries from ChatGPT-4 (3.0 [0]; *P* = .07), ChatGPT-4o (3.0 [0]; *P* = .39), and Llama 3.3-70B (3.0 [0]; *P* = .39) were statistically similar. Responses from Llama 3.3-70B were assessed as being slightly less relevant (3.0 [0] vs 3.0 [0]; *P* = .01) than those from human controls, while those from ChatGPT-4o were slightly less fluent (3.0 [0.5] vs 3.0 [0]; *P* < .01). Other scores were statistically similar between the models and the control. The most common errors made by the LLMs were factually incorrect statements, followed by omissions of relevant information (Table 2). The Krippendorf alpha score was 0.69 for accuracy, 0.41 for thoroughness, 0.65 for relevancy, and 0.22 for fluency, suggesting significant interrelater variability.

**Table 1:** Results of Evaluations

Quality	Human	ChatGPT-4	Llama 2-70B	ChatGPT-4o	Llama 3.3-70B	*P* value
Accuracy	3.0 (0)	3.0 (1.0)	2.0 (1.25)	3.0 (0)	3.0 (0)	<.001
Thoroughness	3.0 (1.0)	3.0 (0)	2.0 (1.0)	2.0 (1.0)	3.0 (0.75)	.002
Relevancy	3.0 (0)	3.0 (0)	3.0 (0)	3.0 (0)	3.0 (0)	.025
Fluency	3.0 (0)	3.0 (0.5)	3.0 (0)	3.0 (1.0)	3.0 (0)	.006

The first 3 columns display the median (interquartile range) score in each category for each author type.

**Table 2:** Error Analysis Table

Case	Model	Errors
1	Human	Thoroughness: did not include previous medications tried and reason for failure
	Llama 2	Accuracy: incorrectly states patient is on Lialda at the time of presentation (old medication) Thoroughness: omits that patient is on antibiotics Thoroughness: omits that she is currently on prednisone Thoroughness: omits distribution of IBD
	Llama 3.3	
	ChatGPT-4	Accuracy: states she is on a prednisone dose of 40 mg when she is on a taper and the exact dose of her prednisone is not specified Accuracy: states patient did not tolerate Lialda, when it was merely ineffective
	ChatGPT-4o	
2	Human	
	Llama 2	Accuracy, thoroughness: incorrectly states that the date of disease onset is not specified Thoroughness: omitted information about recent MRE, colonoscopy, relevancy: hepatitis steatosis is not as relevant to IBD assessment
	Llama 3.3	
	ChatGPT-4	Fluency: "with no issues in the terminal ileum"
	ChatGPT-4o	
3	Human	
	Llama 2	Accuracy: incorrectly states patient underwent recent flexible sigmoidoscopy Thoroughness: omits history of anal fissures
	Llama 3.3	
	ChatGPT-4	Accuracy: incorrectly names psyllium, polyethylene glycol, and linaclotide as IBD medications
	ChatGPT-4o	Thoroughness: omits history of anal fissures
4	Human	
	Llama 2	Accuracy: incorrectly states that there is no information or date on the most recent flexible sigmoidoscopy Accuracy: incorrectly states that current disease activity is not specified
	Llama 3.3	
	ChatGPT-4	Fluency: unusual phrasing, “in her disease journey”
	ChatGPT-4o	Thoroughness: omitted date of and information from most recent endoscopic procedure Thoroughness: omitted history of pouchitis
5	Human	Thoroughness: omitted date of disease onset, prior IBD medications, and distribution of IBD
	Llama 2	Accuracy: incorrectly states that the date and results of endoscopic procedures are not specified. Accuracy: incorrectly states he has had EGD and colonoscopies before when he has only had a sigmoidoscopy Accuracy: incorrectly states he is taking ciprofloxacin
	Llama 3.3	Accuracy: states patient has severe IBD activity, when it seems more likely that the patient has pouchitis
	ChatGPT-4	
	ChatGPT-4o	Accuracy: inaccurately states that patient has been on ciprofloxacin since 2022
6	Human	Thoroughness: omitted date and findings from most recent endoscopy and imaging and omitted prior IBD medication history
	Llama 2	Accuracy: incorrectly states he has not had prior endoscopy; accuracy: incorrectly states that there is no information about disease activity available
	Llama 3.3	
	ChatGPT-4	Accuracy: incorrectly states infliximab caused abdominal pain
	ChatGPT-4o	Thoroughness: omitted specifics of endoscopy and MRE findings, summarizing as showing “chronic inflammation and ulceration”
7	Human	Thoroughness: likely typo but did not explicitly specify that patient has Crohn’s disease
	Llama 2	Accuracy: incorrectly states patient has not had prior colonoscopy
	Llama 3.3	
	ChatGPT-4	
	ChatGPT-4o	Fluency: unusual phrasing, “She has been on adalimumab since 2017, achieving clinical remission with no reported failures or disease flare-ups”
8	Human	
	Llama 2	
	Llama 3.3	Thoroughness: omitted details of treatment with Entyvio
	ChatGPT-4	
	ChatGPT-4o	
9	Human	
	Llama 2	Accuracy: inaccurately states patient has a history of medication nonadherence when she was only lost to follow-up Thoroughness: omits date of most recent colonoscopy
	Llama 3.3	
	ChatGPT-4	
	ChatGPT-4o	
10	Human	
	Llama 2	Incorrectly states date of last sigmoidoscopy was May 2023 instead of April 2023 Incorrectly states patient has only received 1 induction dose of infliximab
	Llama 3.3	
	ChatGPT-4	
	ChatGPT-4o	
11	Human	
	Llama 2	Accuracy: incorrectly states she has never undergone any endoscopic procedures before Thoroughness: omits distribution of IBD
	Llama 3.3	
	ChatGPT-4	
	ChatGPT-4o	

Few studies have evaluated the performance of LLMs when applied to clinical summarization tasks, and to our knowledge, this is the first in the domain area of IBD. One group has evaluated LLMs in the context of summarizing hepatology referral documents.^1^ There have also been evaluations of LLMs in medical education,^5^ patient education about endoscopic procedures,^6^ extracting relevant data from clinical guidelines,^7^ and identifying the medical records of patients who experienced gastrointestinal bleeding from an electronic medical record.^2^ In most of these applications, LLMs performed comparably to humans. However, as our research demonstrates, conclusions about their performance are not always generalizable across medical tasks, different specialty areas, or even between models of similar sizes.

This was a preliminary study with a small sample size. It may be underpowered to detect more subtle differences in performance across models. Cost and computing capacity limited our evaluation to using open-source models with 70 billion parameters; larger models may behave differently. Performance may differ with fine-tuned models or more advanced prompt engineering techniques, such as chain-of-thought reasoning, ReACT, and Reflexion, which are underexplored in clinical contexts.^8^^,^^9^ Another significant limitation of this study was high interrater variability, which highlights the subjective nature of assessment of digital summarization tasks. We attempted to address this with the use of a detailed rubric, 2 raters, and blinding. Future research could employ more raters or incorporate more extensive training for each rater, for example, pretask training session, instructional examples, and counterexamples.^10^

This study demonstrates that both commercial and open-source LLMs show promise for IBD clinical text summarization. Extraction and synthesis of relevant data from medical charts are functions that practicing clinicians perform constantly, which may be suitable for augmentation with LLMs. Future research is necessary to evaluate feasibility with real-world clinical data and clinical workflows.

## Supplementary Materials

Supplementary Figures 1–3 and Supplementary Table

Extended PDF

## References

[1] Shroff H. (2026). Am J Gastroenterol.

[2] Zheng N.S. (2025). Gastroenterology.

[3] Maaz S. (2024). Front Med.

[4] Croxford E. (2025). Npj Health Syst.

[5] Kanjee Z. (2023). JAMA.

[6] Tariq R. (2024). Gastroenterology.

[7] Ge J. (2024). Hepatology.

[8] Prompt engineering guide. https://www.promptingguide.ai/.

[9] Gallifant J. (2025). Nat Med.

[10] Tam T.Y.C. (2024). NPJ Digit Med.

