# Supplementary material for: Summarization of Narrative Clinical Data of Inflammatory Bowel Disease With Foundational Large Language Models
Source: Gastro Hep Adv. 2026 Apr 13;5(7):100967. doi: 10.1016/j.gastha.2026.100967 (PMC13223828; doi:10.1016/j.gastha.2026.100967)
Supplement: Supplementary Figures 1–3 and Supplementary Table [file mmc1.pdf]

Supplemental Data

Figure 1. Prompts used

**Step**	**Input**
**1**	Extract the variables below from the input text. If the variable cannot be found, the value should be "not defined".<br><br>Variables:<br>Age<br>Gender<br>Distribution of IBD<br>Date of disease onset<br>List previous surgeries the patient has had<br>List of IBD medications patient is currently on<br>List any previous medications for IBD the patient took, and if the patient failed the medication, the reason why they failed.<br>The current activity of the patient's IBD (well-controlled, active with mild symptoms, moderately active, severely active)<br>Date and results of most recent flexible sigmoidoscopy, EGD/esophagogastroduodenoscopy, and colonoscopies if any.<br><br>Input Text:

**2**	Take the variables below and generate a concise, precise, one paragraph assessment of the patient's disease that includes all the variables listed and tries to limit extraneous information.<br><br>*[Output from previous prompt]*

Figure 2. Rubric Used by Graders

	0	1	2	3
**Accuracy**	There were three discrepancies between the HPI and assessment	There were two discrepancies between the HPI and assessment	There was one discrepancy between the HPI and assessment	The assessment was completely accurate. There were no discrepancies between the HPI and assessment.
**Thoroughness**	One or more details that are critical for medical decision-making were missing in the assessment.	Multiple (3 or more) minor details I would expect in an assessment were missing, but no critical details were missing.	One or two minor details that I would expect in an assessment were missing	Every clinically significant detail I would expect in the assessment was included.
**Relevancy**	There were many (>3) extraneous details included that were not necessary for clinical care.	There were 2-3 extraneous details included that were not necessary for clinical care.	There was 1 extraneous detail that was not necessary for clinical care.	Every detail included was medically relevant.
**Fluency**	Most of the paragraph is incomprehensible. This does not sound like a fluent user of the language.	There are multiple (3 or more) phrases that are awkwardly constructed, but it is somewhat comprehensible.	Everything is comprehensible. There may be one or phrase that is awkwardly constructed.	Everything is comprehensible and easily readable. There are no phrases that are awkwardly constructed.

Table 1. Performance Scores for all Models

Case	LLM	Accuracy	Thoroughness	Relevancy	Fluency	Reviewer
1	ChatGPT-4o	3	3	3	2	1
1	ChatGPT-4o	3	3	3	3	2
1	ChatGPT-4	2	3	3	3	1
1	ChatGPT-4	1	3	3	3	2
1	Human	3	2	3	3	1
1	Human	3	2	3	3	2
1	Llama3.3	3	3	3	3	1
1	Llama3.3	3	3	3	3	2
1	Llama2.2	2	2	3	3	1
1	Llama2.2	2	2	3	3	2
2	ChatGPT-4o	3	2	3	2	1
2	ChatGPT-4o	2	3	3	3	2
2	ChatGPT-4	3	3	3	3	1
2	ChatGPT-4	3	3	3	2	2
2	Human	3	3	3	3	1
2	Human	3	3	3	3	2
2	Llama3.3	3	3	3	3	1
2	Llama3.3	3	3	3	3	2
2	Llama2.2	2	2	3	3	1
2	Llama2.2	3	2	2	3	2
3	ChatGPT-4o	3	2	3	3	1
3	ChatGPT-4o	1	2	3	3	2
3	ChatGPT-4	1	3	3	3	1
3	ChatGPT-4	1	3	3	3	2
3	Human	2	3	3	3	1

3	Human	3	3	3	3	2
3	Llama3.3	3	3	3	3	1
3	Llama3.3	1	3	3	3	2
3	Llama2.2	2	2	3	3	1
3	Llama2.2	2	2	3	3	2
4	ChatGPT-4o	3	2	3	3	1
4	ChatGPT-4o	3	1	3	3	2
4	ChatGPT-4	3	3	3	2	1
4	ChatGPT-4	3	3	3	2	2
4	Human	3	3	3	3	1
4	Human	3	3	3	3	2
4	Llama3.3	3	3	3	3	1
4	Llama3.3	2	3	3	3	2
4	Llama2.2	1	2	3	3	1
4	Llama2.2	1	3	3	3	2
5	ChatGPT-4o	2	1	3	2	1
5	ChatGPT-4o	2	3	3	3	2
5	ChatGPT-4	3	3	3	3	1
5	ChatGPT-4	3	3	3	3	2
5	Human	3	1	3	3	1
5	Human	3	2	3	3	2
5	Llama3.3	1	3	3	3	1
5	Llama3.3	2	2	3	3	2
5	Llama2.2	0	2	3	3	1
5	Llama2.2	0	3	3	3	2
6	ChatGPT-4o	3	1	3	2	1
6	ChatGPT-4o	3	2	3	3	2

6	ChatGPT-4	2	3	3	3	1
6	ChatGPT-4	2	2	3	3	2
6	Human	3	1	3	3	1
6	Human	3	1	3	3	2
6	Llama3.3	3	2	3	3	1
6	Llama3.3	2	3	3	3	2
6	Llama2.2	0	0	3	3	1
6	Llama2.2	0	0	3	3	2
7	ChatGPT-4o	3	3	3	2	1
7	ChatGPT-4o	1	3	3	2	2
7	ChatGPT-4	3	3	3	3	1
7	ChatGPT-4	3	3	3	3	2
7	Human	1	0	3	3	1
7	Human	3	0	3	3	2
7	Llama3.3	3	3	3	3	1
7	Llama3.3	3	2	3	3	2
7	Llama2.2	3	2	3	3	1
7	Llama2.2	2	3	3	3	2
8	ChatGPT-4o	3	1	3	3	1
8	ChatGPT-4o	3	3	3	3	2
8	ChatGPT-4	2	3	3	3	1
8	ChatGPT-4	2	3	3	3	2
8	Human	3	3	3	3	1
8	Human	3	3	3	3	2
8	Llama3.3	3	1	3	3	1
8	Llama3.3	3	1	3	3	2
8	Llama2.2	3	3	3	3	1

8	Llama2.2	3	3	3	3	2
9	ChatGPT-4o	3	1	3	3	1
9	ChatGPT-4o	3	3	3	3	2
9	ChatGPT-4	3	3	3	3	1
9	ChatGPT-4	3	2	3	3	2
9	Human	3	3	3	3	1
9	Human	3	3	3	3	2
9	Llama3.3	3	1	3	2	1
9	Llama3.3	3	3	2	3	2
9	Llama2.2	2	2	3	3	1
9	Llama2.2	1	2	3	3	2
10	ChatGPT-4o	3	3	3	2	1
10	ChatGPT-4o	3	2	3	3	2
10	ChatGPT-4	3	3	3	3	1
10	ChatGPT-4	3	2	3	3	2
10	Human	3	3	3	3	1
10	Human	3	3	3	3	2
10	Llama3.3	3	3	2	2	1
10	Llama3.3	3	3	2	3	2
10	Llama2.2	2	2	3	3	1
10	Llama2.2	1	2	3	3	2
11	ChatGPT-4o	3	3	3	3	1
11	ChatGPT-4o	3	2	3	3	2
11	ChatGPT-4	3	3	3	3	1
11	ChatGPT-4	3	3	3	3	2
11	Human	3	3	3	3	1
11	Human	3	3	3	3	2

11	Llama3.3	3	3	2	2	1
11	Llama3.3	3	3	3	3	2
11	Llama2.2	0	3	3	3	1
11	Llama2.2	0	0	3	3	2

Figure 3. Box Plots with Jittered Data Points Summarizing Scores

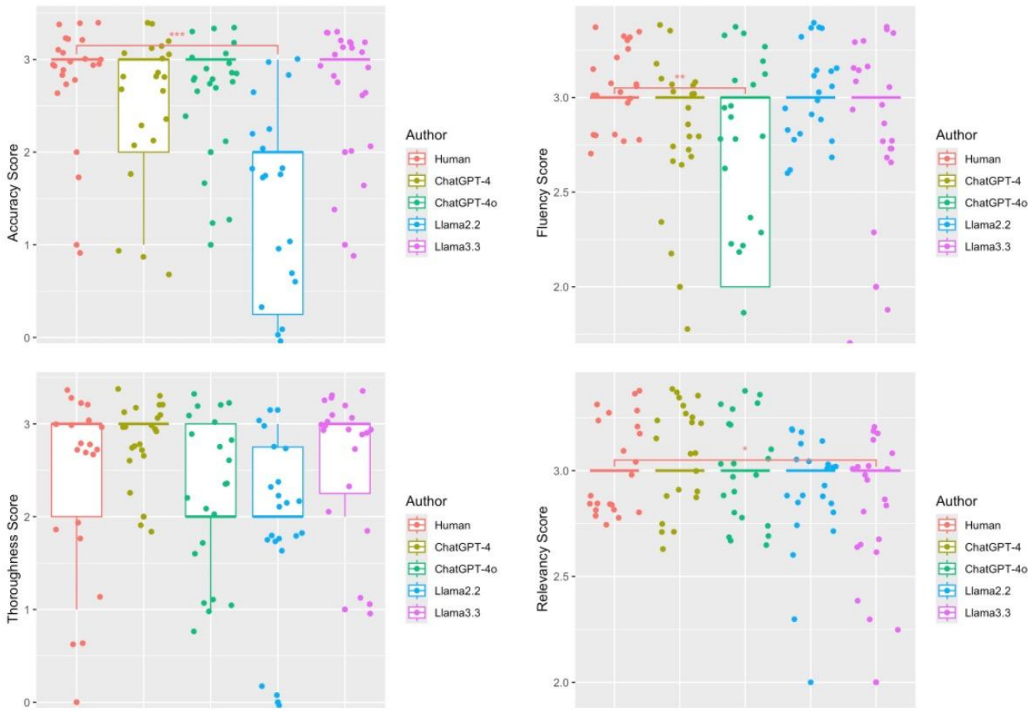

Note:

All mock progress note text and generated summary outputs used in this research project can be found online under an open-source MIT license for further research or review at: https://github.com/soonwook/suppl_llm_summarization_project
